# MicroRNA-455 suppresses the oncogenic function of HDAC2 in human colorectal cancer

**DOI:** 10.1590/1414-431X20176103

**Published:** 2017-05-18

**Authors:** Q.D. Mao, W. Zhang, K. Zhao, B. Cao, H. Yuan, L.Z. Wei, M.Q. Song, X.S. Liu

**Affiliations:** 1 Department of Gastroenterology, Huangdao Division, The Affiliated Hospital of Qingdao University, Qingdao, China; 2 Department of Gastroenterology, Laoshan Division, The Affiliated Hospital of Qingdao University, Qingdao, China

**Keywords:** Colorectal cancer, Histone deacetylase 2, miR-455, Proliferation, Apoptosis

## Abstract

Colorectal cancer (CRC) is the fourth leading cause of cancer-induced mortality. Histone deacetylase 2 (HDAC2) is involved in prognosis and therapy of CRC. This study aimed to explore novel therapeutic targets for CRC. The alteration of HDAC2 expression in CRC tissues was estimated by qRT-PCR. After lentivirus transfection, HDAC2 knockdown was confirmed by western blot analysis. The effect of HDAC2 knockdown on cell proliferation was then assessed by 3-(4,5-dimethylthiazol-2-yl)-2, 5-diphenyltetrazolium bromide (MTT) assay. Screened by TargetScan, microRNA (miR)-455 was predicted to bind to 3′UTR of HDAC2 and the prediction was verified by luciferase assay. Finally, cells were transfected, respectively, with miR-455 mimics or miR-455 negative control (miR-NC) and the expression of HDAC2, cell proliferation and apoptosis of transfected cells were respectively evaluated by western blot analysis, MTT assay and flow cytometry. Results showed that the HDAC2 expression was up-regulated in CRC tissues (P<0.05). HDAC2 knockdown significantly decreased cell viability at day 3 (P<0.05), day 4 (P<0.01), and day 5 (P<0.001) after infection. Then, miR-455 was verified to directly target HDAC2, resulting in a significant difference in luciferase activity (P<0.01). Moreover, miR-455 decreased the expression of HDAC2 (P<0.01). miR-455 remarkably decreased cell viability at day 3 (P<0.05), day 4 (P<0.01), and day 5 (P<0.001) after transfection while inducing cell apoptosis (P<0.001). In conclusion, miR-455 inhibited cell proliferation while inducing cell apoptosis by targeting HDAC2 in CRC cells.

## Introduction

Colorectal cancer (CRC) is reported to be the third most common cancer with 1.2 million new cases globally, as well as the fourth leading cause of cancer mortality, accounting for 600 thousand deaths every year (1,2). Unlike other cancers, the risk factors for CRC are multiple, including family history of CRC, smoking, high consumption of red meat, etc. (3–5). The incidence of CRC is strongly related to age, presenting low incidence in people younger than 50 years and high incidence in aging people (6). Accumulative evidence is suggestive of a rapid increase in incidence as well as mortality rates of CRC in low-income countries (7,8). Despite the slow improvement on prognosis of patients with CRC, 5-year relative survival is less than 50% in low-income countries (9,10). Therefore, it is urgent to explore novel and effective therapeutic targets for CRC treatment.

Histone deacetylase 2 (HDAC2), class I of HDACs, which repress gene expression by removing acetyl groups from histone substrates, is a crucial factor in health and disease (11). Overexpression of HDAC2 is proved to confer oncogenic potential to human lung cancer cells by deregulating apoptosis-associated proteins (12). Another study also validated that decrease of HDAC2 could induce cell apoptosis and inhibit cell proliferation in laryngeal squamous cell carcinoma cells (13). Recent clinical results are indicative of associations between HDAC2 and cancer prognosis (14). In terms of CRC, Zhu et al. (15) showed that HDAC2 acted as a potential target in CRC therapy. An additional research also implied that selective interference with HDAC2 could induce multiple effects on cell death signaling in CRC cells (16). As a consequence, expression level of HDAC2 is pivotal for prognosis evaluation and therapy of CRC.

MicroRNAs (miRNAs), which are single-stranded RNA molecules with approximately 22 nucleotides in length, bind to target sites located at 3′-untranslated region (3′UTR) of target mRNAs to induce gene silencing (17). Deregulation of miRNAs has been applied in repression of multiple protein expressions. As for HDAC2, miR-21 has been reported to down-regulate expression of HDAC2 (18). miR-145 also acts as a tumor suppressor by inhibition of HDAC2 in liver cancer (19). Considering the effects of HDAC2 on cell proliferation and apoptosis in cancer cells, we focused on the specific miRNA, which directly targeted HDAC2, to explore the potential therapeutic method for CRC. Moreover, the effects of the specific miRNA on cell proliferation and apoptosis were further studied. This exploration may provide a novel therapeutic method for CRC treatment.

## Material and Methods

### Patients and tissue samples

Clinical tissue samples were obtained from 20 patients (10 men and 10 women, average age 59.15±10.06 years) who were diagnosed with CRC from March 2014 to December 2014. The tumor and adjacent non-tumor tissues were surgically resected and collected in separate tubes. After snap-frozen by liquid nitrogen, the tissues were stored at –80°C for further study. The non-tumor tissues were obtained from the distal edge of the resection (≥10 cm from the carcinoma). Samples were collected after receiving the written informed consent from the patients. The investigation was approved by the institutional Ethics Committee of the Affiliated Hospital of Qingdao University.

### Quantitative reverse transcription PCR (qRT-PCR)

Tissue samples were treated with TRIzol reagent (Invitrogen, USA) and total RNA was extracted according to the manufacturer's instructions. The cDNA was reversely transcribed by using GoScript^™^ Reverse Transcriptase (Promega, USA) in line with supplier's instructions. qPCR was performed according to the manufacturer's protocol of Power SYBR-Green Master Mix (Bio-Rad, USA). Primes were designed and synthesized as follows: HDAC2: forward 5′-GCT ATT CCA GAA GAT GCT GTT C-3′, reverse 5′-GTT GCT GAG CTG TTC TGA TTT G-3′; β-tubulin: forward 5′-CGT GTT CGG CCA GAG TGG TGC-3′, reverse 5′-GGG TGA GGG CAT GAC GCT GAA-3′ (GenePharma, China). The relative expression level was calculated using the 2^-ΔΔCt^ method (20) and normalized to β-tubulin.

### Cell culture

Human colon cancer HCT116 cells were purchased from American Type Culture Collection (USA) and maintained in McCoy's 5A medium (Gibco, USA) containing 10% fetal bovine serum (FBS, Gibco) and 100 U/mL penicillin/streptomycin (Invitrogen, USA). Cells were incubated in a humidified incubator with 5% CO_2_ at 37°C.

### Lentivirus transfection

Lentivirus (GV118) encoding short hairpin RNA (shRNA) targeting HDAC2 (HDAC2 shRNA) and its negative control (shRNA-NC) were both prepared by GenePharma. HCT116 cells were seeded on 6-well plates with a density of 10^4^ cells/well and maintained for 12 h at 37°C. Then, the medium was replaced by McCoy's 5A medium. With a multiplicity of infection (MOI) of one, 2 μL of shRNA was added into each well. Following incubation for 12 h at 37°C, the medium was replaced by fresh McCoy's 5A medium again. The cells were harvested for western blot analysis to assess the knockdown efficiency after being cultured for 96 h at 37°C.

### miRNA transfection

HCT116 cells were seeded on 6-well plates with a density of 10^4^ cells/well. When the cells reached 70% confluence, 100 pmol of miR-455 mimics or its negative control (miR-NC; both from Genepharma) was added to each well accompanied by Lipofectamine 2000 (Invitrogen, USA). The cells were harvested for further study after being cultured for 48 h at 37°C.

### Luciferase activity assay

After prediction by TargetScan, the 3′UTR fragment of HDAC2 binds to miR-455. Thus, the 3′UTR segment of the HDAC2 gene, containing putative binding site for miR-455, was inserted into pmirGLO vector (Promega). After sequencing, the vector containing 3′UTR of HDAC2 was co-transfected with miR-455 mimics or miR-NC. Luciferase activity was assayed at 48 h post-transfection, according to the manufacturer's instructions of Dual-Luciferase Reporter Assay System (Promega).

### Western blot analysis

The transfected HCT116 cells were washed by phosphate-buffered saline (PBS) and then were treated according to the manufacturer's protocol of RIPA lysis buffer (Beyotime, China). After measurement by BCA assay kit (Pierce, USA), equal amounts of proteins were loaded and separated by 12% sodium dodecyl sulfate polyacrylamide gel electrophoresis (SDS-PAGE). The proteins in gel were transferred to the polyvinylidene fluoride membranes (Millipore, USA), followed by blocking with 5% skim milk (Nestlé, China). Immunoblotting was carried out with primary antibodies against HDAC2 (sc-81599) or β-tubulin (sc-5274) (both from Santa Cruz Biotechnology, USA) at 4°C overnight. Then, the membranes were incubated with horseradish peroxidase-marked rabbit anti-mouse IgG antibody at room temperature for 1 h. Finally, the proteins were detected by ECL Plus western blotting detection kit (Amersham Pharmacia Biotech, Germany) in line with manufacturer's instructions. The bands were analyzed with Image Lab™ software (Bio-Rad). The relative expression level was calculated by 2^-ΔΔCt^ method (21) and normalized by β-tubulin.

### Cell viability assay

The transfected cells were placed, in triplicate, in a 96-well plate with a density of 1.5×10^3^ cells/well. The cells were cultured at 37°C for 5 days. At day 1, 2, 3, 4, or 5, 10 μL of 3-(4,5-dimethylthiazol-2-yl)-2,5-diphenyltetrazolium bromide (MTT, 5 mg/mL, Sigma, USA) was added to each well and the mixture was incubated for 4 h at 37°C. Then, 100 μL of HCl (0.04 N in 2-propanol) was added to each well with thorough mixing. The absorbance was read at wavelengths of 570 nm and 650 nm (background reading subtracted) with a microplate reader (Bio-Rad).

### Apoptosis assay

After transfection, the HCT116 cells were harvested and diluted to 3×10^6^ with binding buffer of FITC Annexin V/Dead Cell Apoptosis Kit (Invitrogen). According to the manufacturer's instructions, the cells were stained and then analyzed by flow cytometer (BD Biosciences, Germany). The results were analyzed using FlowJo 10.0.7 software (Treestar Inc., USA).

### Statistical analysis

All experiments were repeated three times. The results are reported as means±SD. Statistical analysis was performed using Graphpad Prism 5 software (GraphPad, USA). Two-tailed unpaired *t*-test was employed to calculate the P-values. P<0.05 was considered to be statistically significant.

## Results

### HDAC2 expression was up-regulated in human CRC tissues

**Figure 1.**
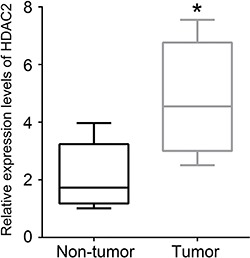
Expression of HDAC2 in human CRC tissues was higher than that in neighboring non-tumor colon tissues. Data are reported as the median of 20 independent experiments. Error bars indicate minimum and maximum. *P<0.05 (two-tailed unpaired *t*-test). HDAC2: histone deacetylase 2; CRC: colorectal cancer.

As shown in Figure 1, the mRNA expression level of HDAC2 in CRC tissues was markedly enhanced compared with non-tumor tissues (P<0.05).

### HDAC2 knockdown inhibited the proliferation of HCT116 cells

**Figure 2.**
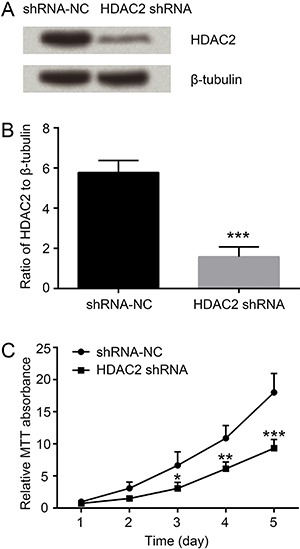
Knockdown of HDAC2 reduced cell viability in HCT116 cells. Cells were infected with shRNA-NC or HDAC2 shRNA. *A* and *B*, Protein expression of HDAC2 in infected cells. *C*, Cell viability of infected cells. Data are reported as the mean±SD of 3 independent experiments. *P<0.05; **P<0.01; ***P<0.001 (two-tailed unpaired *t*-test). HDAC2: histone deacetylase 2; HDAC2 shRNA: short hairpin RNA targeting HDAC2; shRNA-NC: negative control of HDAC2 shRNA; MTT: 3-(4,5-dimethylthiazol-2-yl)-2,5-diphenyltetrazolium bromide.

To validate the effect of HDAC2 on cell viability of HCT116 cells, we used lentivirus infection to realize HDAC2 knockdown. In Figure 2A and B, the protein expression level of HDAC2 in cells infected with HDAC2 shRNA was significantly reduced compared with cells infected with shRNA-NC (P<0.001), indicating HDAC2 was successfully silenced. The subsequent MTT assay performed in infected cells showed that HDAC2 knockdown remarkably decreased cell viability, resulting in significant difference at 3 days (P<0.05), 4 days (P<0.01), and 5 days (P<0.001) compared with cells infected with shRNA-NC (Figure 2C). Thus, we concluded that HDAC2 knockdown inhibited cell proliferation of HCT116 cells.

### miR-455 directly targeted HDAC2 in HCT116 cells

**Figure 3.**
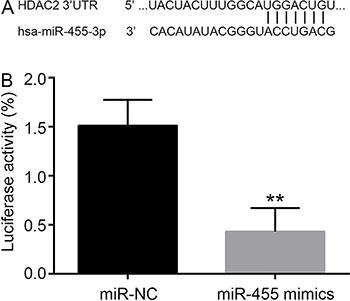
miR-455 directly targeted HDAC2 in HCT116 cells. *A*, Diagram showing miR-455 formed base-pair with the 3′ UTR of HDAC2. *B*, Luciferase activity of cells co-transfected with pmirGLO vector containing 3′UTR segment of the HDAC2 and miR-NC or miR-455 mimics. Data are reported as the mean±SD of 3 independent experiments. **P<0.01 (two-tailed unpaired *t*-test). HDAC2: histone deacetylase 2; 3′UTR: 3′-untranslated region; miR-455 mimics: microRNA-455 mimics; miR-NC: negative control of miR-455.

Screened by ScanTarget, miR-455 was predicted to bind to 3′UTR of HDAC2 (Figure 3A). After co-transfection of pmirGLO vector containing segmental 3′UTR of HDAC2 and miR-NC or miR-455 mimics in HCT116 cells, the luciferase activity was assayed and results suggested that co-transfection with miR-455 mimics significantly reduced the luciferase activity compared with cells co-transfected with miR-NC (P<0.01, Figure 3B), indicating that miR-455 directly targeted HDAC2 in HCT116 cells.

### Ectopic expression of miR-455 reduced the expression of HDAC2 in HCT116 cells

**Figure 4.**
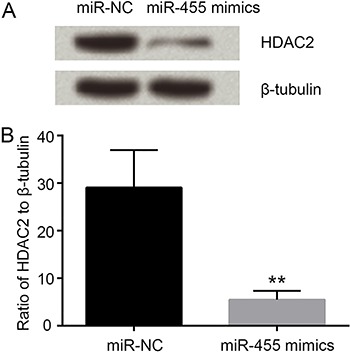
miR-455 reduced the expression of HDAC2. Data are reported as the mean±SD of 3 independent experiments. **P<0.01 (two-tailed unpaired *t*-test). HDAC2: histone deacetylase 2; miR-455 mimics: microRNA-455 mimics; miR-NC: negative control of miR-455.

miR-NC and miR-455 mimics were respectively transfected into HCT116 cells, followed by western blot analysis. The protein expression level of HDAC2 was obviously reduced by transfection of miR-455 mimics in comparison with cells transfected with miR-NC (P<0.01, Figure 4A and B). The data suggested that miR-455 overexpression inhibited HDAC2 expression.

### Ectopic expression of miR-455 inhibited cell proliferation and induced cell apoptosis of HCT116 cells

**Figure 5.**
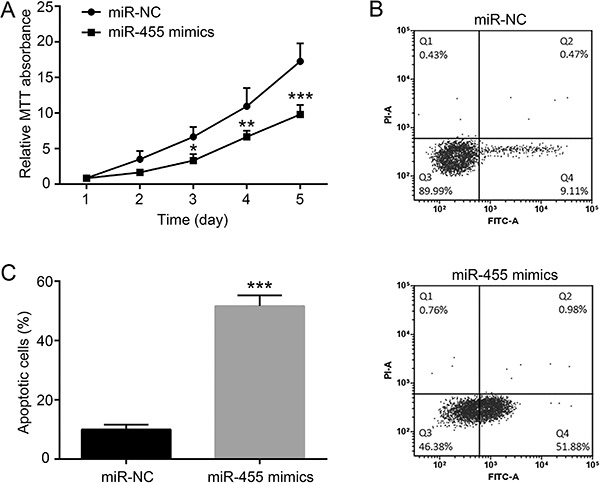
miR-455 inhibited proliferation and induced apoptosis of HCT116 cells. Cell viability (*A*) and cell apoptosis (*B* and *C*) of cells transfected with miR-NC or miR-455 mimics were measured by MTT assay and flow cytometry, respectively. Data are reported as the mean±SD of 3 independent experiments. *P<0.05; **P<0.01; ***P<0.001 (two-tailed unpaired *t*-test). MTT: 3-(4,5-dimethylthiazol-2-yl)-2,5-diphenyltetrazolium bromide; HDAC2: histone deacetylase 2; miR-455 mimics: microRNA-455 mimics; miR-NC: negative control of miR-455.

Cell viability was markedly decreased by miR-455 mimics, resulting in significant difference at 3 (P<0.05), 4 (P<0.01), and 5 days (P<0.001) compared with cells infected with miR-NC (Figure 5A). At the same time, cell apoptosis was remarkably increased by miR-455 mimics compared with cells transfected with miR-NC (P<0.001, Figure 5B-C). Therefore, we drew a conclusion that ectopic expression of miR-455 inhibited cell proliferation while inducing cell apoptosis of HCT116 cells.

## Discussion

Our observations suggested that miR-455 suppressed the oncogenic function of HDAC2 in human CRC. We found that the expression level of HDAC2 was significantly enhanced in CRC tissues. Then, MTT assay of lentiviral infected cells demonstrated that HDAC2 knockdown obviously inhibited cell proliferation. Screened by TargetScan, miR-455 was predicted to bind to 3′UTR of HDAC2 and the prediction was further confirmed by luciferase assay. Finally, miR-455 was proved to effectively inhibit cell proliferation while inducing cell apoptosis in CRC cells.

HDAC2 has been reported to be aberrantly expressed in diverse cancers (22). Weichert et al. (23) demonstrated that HDAC2 was highly expressed in prostate cancer. The expression of HDAC2 was found to be increased in human hepatocellular carcinoma and CRC (24,25). In the experiments by Stypula-Cyrus et al. (26), HDAC2 was even selected as a biomarker for CRC. The results in our study on clinical CRC tissues were similar to previous conclusions (23-26), implying that therapeutic strategies for CRC targeting HDAC2 should be effective.

The development of CRC is strongly associated with imbalance between cell proliferation and apoptosis (27). A novel HDAC2 inhibitor, termed 4SC-202, has been found to inhibit cell survival and proliferation while promoting cell apoptosis in human CRC cells (28). Thus, we constructed a lentiviral vector to silence HDAC2 and thereby explored the effect of HDAC2 suppression on cell proliferation in HCT116 cells. The results validated that HDAC2 knockdown could suppress cell proliferation of HCT116 cells, which was consistent with findings in breast cancer, in which the knockdown of HDAC2 has been proven to inhibit cell proliferation by increasing the binding activity of p53 (29). Therefore, HDAC2 suppression plays important roles in the tumorigenesis and development of CRC and might become a novel therapeutic target.

Currently, there are large numbers of anti-tumor agents that might generate drug resistance after long-term use, along with intrinsic toxicity against normal cells (27). The drawbacks of current anti-tumor agents highlight the urgent need for the development of novel CRC treatment. In our present study, we focused on miRNA which functions through degradation or transcription inhibition of target genes. By bioinformatics methods, we found the miR-455 might specifically bind to 3′UTR of HDAC2 gene. miR-455, which locates at the protein-coding gene Col27a1, has been reported to be involved in early chondrogenic differentiation regulation (30), down-regulation of MMP-9 during exercise (31), hypoxia signaling (32) and acquired temozolomide resistance in glioblastoma cells (33). However, investigations focused on the association between miR-455 and CRC are very few. As of now, there is only one report that demonstrated the implication of miR-455 in cell proliferation and invasion of CRC cells by targeting RAF proto-oncogene serine/threonine-protein kinase (34). Therefore, the study on associations between miR-455 and CRC might contribute to expand the current knowledge of miR-455. Besides, miR-455 might be practicable for treatment of CRC.

HDAC2 was validated as a target gene of miR-455 by luciferase assay in HCT116 cells co-transfected with HDAC2 3′UTR and miR-455 mimics or miR-NC. The subsequent experiments on protein expression level of HDAC2 in HCT116 cells transfected with miR-NC or miR-455 mimics verified the effective repression of miR-455 on HDAC2 expression. Also, the cell proliferation and apoptosis assays performed in cells transfected with miR-NC or miR-455 mimics successfully corroborated that the repression of HDAC2 expression induced by miR-455 obviously inhibited cell proliferation while promoted cell apoptosis in HCT116 cells. In other words, miR-455 could effectively repress cell proliferation while inducing cell apoptosis via targeting HDAC2.

In conclusion, HDAC2 was up-regulated in CRC tumor tissues and down-regulated HDAC2 significantly inhibited cell proliferation in CRC cells. miR-455 directly targeted HDAC2 and its overexpression suppressed cell proliferation and induced cell apoptosis in CRC cells through HDAC2 repression. The present study not only expanded the knowledge of miR-455 but also provided a novel therapeutic target for CRC.
